# Oral health and oral health needs among patients with serious mental illness: reflections and experiences of psychiatric staff in Sweden

**DOI:** 10.1186/s40359-025-02780-3

**Published:** 2025-04-28

**Authors:** Charlotte Johansson, Linda Beckman, Ingrid Rystedt, Ann-Catrin André Kramer, Ulrika Lindmark

**Affiliations:** 1 Department of Health Science, Karlstad University, Karlstad, SE 651 88 Sweden; 2 Department of Health Service Research, Management and Policy, University of Florida, Florida, FL 32603 USA; 3 Department of Health, Medicine and Caring Sciences, Linköping University, Linköping, SE 581 83 Sweden

**Keywords:** Dental health, Integrated care, Health promotion, Mental health, Comorbidity, Qualitative research

## Abstract

**Background:**

Due to organizational structures, there is a gap between psychiatric and dental care services that limits collaboration and knowledge sharing, and this can have a negative impact on patients’ oral health or general health and vice versa. To facilitate the integration between dentistry and psychiatry, more insight and knowledge is needed on psychiatric staffs’ work with patients’ oral health. Therefore, the aim of this study was to explore the experiences of psychiatric staff regarding patients’ oral health and meeting their oral health needs.

**Methods:**

Using a semi-structured guide, staff (*n* = 13) in Swedish psychiatric and forensic settings and in municipal housing support services were interviewed about their experiences with patient oral health. The interviews took place between April 2022 and June 2023. The data were analysed with inductive qualitative content analysis.

**Results:**

This study identified challenges such as organizational issues, complex administrations, and a lack of tools for integrating oral health into psychiatric care. The patients perceived to face daily health-related challenges, and the informants identified opportunities to include oral health into tools for health promotion and the facilitation of healthy lifestyle changes.

**Conclusion:**

Psychiatric staff possess central knowledge and insight into the life situations of patients with severe mental illness, and psychiatric staff consider dental staff to be key partners. Collaboration between psychiatric and dental staff is essential for developing strategies to integrate oral health perspectives into current screening and psychopedagogical models and practices.

**Supplementary Information:**

The online version contains supplementary material available at 10.1186/s40359-025-02780-3.

## Background

An organizational gap between psychiatric and dental services has been identified [1–4]. Today there is a better understanding of the complexity of care needs in both psychiatry and dentistry; however, knowledge about the links between people’s mental health and oral health and interventions that can improve their oral health is insufficient [5]. In the 1960s, organizational changes occurred within the field of psychiatry in Sweden and other countries, and state institutions were decentralized into outpatient clinics, inpatient clinics, and forensic psychiatric clinics. Compared to the pre-deinstitutionalization era, patients are expected to take responsibility for themselves and to be actively involved in their treatment according to their abilities [6]. Today, approximately 300,000 patients annually are seen in Swedish outpatient psychiatry contexts, dominated by the diagnoses of depression, anxiety and stress-related disorders [7, 8]. Inpatient psychiatry contexts see 15,000- 20,000 patients annually, with primarily the diagnoses of schizophrenia, bipolar disorder, and severe depression [8]. Between 1,000–1,200 patients are under forensic psychiatry care, typically with the diagnoses of psychosis or severe personality disorders [9]. Concurrently, patients with mental illnesses experience barriers in accessing somatic care, including dental care. These barriers include issues of accessibility, adaptation, affordability, and acceptance, resulting in missed opportunities for somatic care. Most such barriers are at a political level [5, 10]. Thus, it is central that there is political will to ensure oral health services are inclusive, accessible, and acceptable to all groups in the population [5, 10]. The Swedish national dental care subsidy, managed by the Social Insurance Agency, provides general and special allowances as well as high-cost protection to provide preventive care and support patients at greater risk of poor oral health. Dentists and hygienists guide patients regarding eligible treatments [11].

The World Health Organisation (WHO) strives to provide comprehensive, integrated, and responsive mental health and social care services in community-based settings [12]. In light of this, it is important to be aware of that some individuals with severe mental illness (SMI) and primarily negative symptoms, tend to neglect personal hygiene, make poor dietary choices, and lack physical activityfurther affecting both their mental and oral health [13–15]. For the development of appropriate support for patients with mental illnesses in regard to their oral health needs, it is important to capitalize on the knowledge and experiences of the staff working in psychiatric health care contexts [16, 17]. From a patient perspective, the stigma around mental health problems persists in society [18], including labelling, stereotyping, social exclusion, and discrimination against those who deviate from the norms [19]. Patients with SMI often have concurrent medical conditions, including oral diseases, which can lead to further stigma. This implies that the oral health of patients with SMI is a responsibility of both psychiatry and dentistry [5, 20, 21].

The WHO identifies oral health as a contributor to overall health, well-being, and quality of life [22]. Oral health includes functions such as speaking, smiling, and chewing without discomfort, and these functions significantly impact general well-being and daily life and social interactions [23]. Oral health problems can, directly or indirectly, arise due to long-term medication, lack of support and advice from dental and psychiatric care and family and relatives, inadequate access to dental care, and financial difficulties [21]. For patients with SMI and comorbid systemic physical conditions such as diabetes or cardiovascular disease, poor oral health can worsen their conditions and vice versa [1, 3, 15].

Healthcare providers often experience communication difficulties both within and across professions, and there is also a lack of coordinated care, insufficient support for patients during dental visits, and long waiting times for receiving care [24, 25]. The Swedish National Board of Health and Welfare (SNBHW) [25] has reported how differences in systems, culture, and leadership hinder effective collaboration. Dental care is generally not integrated into patient-centred processes of care within psychiatric services, probably due to the lack of a structures that facilitate cooperation among providers in the fields of psychiatry and dentistry [5, 26].

Healthcare staff often face challenges in assessing patients' oral health needs due to a lack of strategies for prevention, treatment, and rehabilitation when specific needs are identified. Healthcare staff have reported that patients often resist oral care, with some patients refusing to open their mouths or biting the toothbrush when staff attempt to provide oral care [27]. In addition, patients often report dental fear, which can result in untreated dental issues [28].

Collaboration between psychiatric and dental care staff has many benefits, including training psychiatric staff to assess oral health using checklists, promoting good oral health habits, and ensuring timely referrals to dental care when needed [29–31]. However, there is a need for clearer instructions and education for staff working in psychiatry, as well as more medical knowledge about the oral manifestations of different diseases [26].

It is important to fill knowledge gaps in terms of strategies for identifying and promoting collaboration between psychiatry and dentistry. Patients with SMI are not a homogeneous group, and their functional abilities vary across individuals [5, 29, 32]. Therefore, strategies for psychiatric staff to improve oral health for their patients are needed. These strategies must be comprehensive and must consider individual health needs such as diagnosis, disease severity, housing, and physical disabilities [3, 29]. Psychiatric staff play a crucial role in developing health-promoting models due to their insight into the everyday lives and challenges of patients with SMI, and their experiences can provide valuable guidance for the development of such strategies. Therefore, the aim of this study was to explore the experiences of psychiatric staff regarding patients’ oral health and meeting their oral health needs.

## Methods

### Materials and methods

The current study took an inductive approach with qualitative semi-structured individual interviews with staff employed in various psychiatric service settings, including both inpatient and outpatient services, across Sweden [33]. This study was conducted in alignment with the COREQ 32-item checklist to ensure comprehensive reporting of qualitative methods [34].

### Recruitment and participants

To include a multitude of experiences, the snowballing method was used in the recruitment of informants [35]. The inclusion criterion was staff being employed at a psychiatric clinic in Sweden. The study was presented to psychiatry professionals within the Swedish Association of Local Authorities and Regions (SALAR) [36] and within the network PsykosR – National Quality Register for Psychosis Care in Sweden. After a reflection period, professionals communicated their interest in participating as an informant in the study by email. To expand the opportunity for recruitment, participants were asked to spread knowledge of the study at their respective units around the country. In addition, a recruitment poster was placed in various psychiatric settings, including both psychiatric and forensic psychiatric units, by an administrator in a region in central Sweden. The poster was designed with text and a QR code linked to a video presentation about the study and contact details to indicate interest in participating.

Thirteen volunteers from several regions of Sweden expressed their interest and oral consent in being interviewed within the field of psychiatry, including both licensed and unlicensed personnel (i.e. one physician, five psychiatric nurses, one nurse practitioner, one care aide, one psychologist, one social worker, one occupational therapist, and two municipal housing support aides) and were interviewed (see Supplementary Material) with the aim of exploring experiences of psychiatric professionals regarding the possibilities and challenges in meeting oral health needs among psychiatric patients. The informants responded to demographic survey questions, providing an overview of their years of professional experiences, highest level of education, gender, and age (Table 1).

**Table 1:** Demographics of the study participants

Experiences	Education level	Gender	Age	Interview format	Duration (min)
1–10 years	College/University	M	28–38	Phone	45
1–10 years	College/University	M	28–38	Phone	34
1–10 years	College/University	M	28–38	Phone	30
1–10 years	College/University	F	43–53	Phone	44
1–10 years	College/University	M	58–68	Digital platform	33
1–10 years	Upper secondary school/High school	M	43–53	Phone	47
11–20 years	College/University	F	28–38	Phone	36
11–20 years	Upper secondary school/High school	F	58–68	Phone	35
11–20 years	College/University	F	58–68	Phone	51
11–20 years	College/University	F	58–68	Digital platform	43
11–20 years	College/University	F	58–68	Digital platform	32
21–40 years	College/University	M	58–68	Digital platform	59
21–40 years	Upper secondary school/High school	F	58–68	Digital platform	33
					40

### Data collection and interview guide

Between April 2022 and June 2023, the semi-structured interviews were conducted by the first author, a doctoral candidate as well an experienced dental hygienist and university lecturer, using the digital platforms Zoom or Teams, as well as mobile phone calls. The interviews were audio recorded, stored using the secure infrastructure provided by the university. Each interview session lasted approximately 30 to 60 min with an average of 40 min and aimed to facilitate in-depth discussions. The interview guide was specifically compiled for this study and covered questions related to staff experiences of the possibilities and challenges to enhance overall patient health, with a primary focus on oral health.

The interview guide encompassed topics such as dental care needs among patients with SMI, as well as daily habits and routines, including self-care practices, dietary patterns, beverage consumption, and tobacco use. Prior to the formal recruitment, two pilot interviews were conducted to try and customize the interview guide.The pilot interviews were not integrated into the results of the study, because the questions were adjusted to guide the informants toward the topic. The pilot interviews tended to deviate from the subject.

### Data analysis

The interviews were transcribed verbatim, and qualitative content analysis was utilized at a manifest level using the five-step inductive approach [33]. To gain a comprehensive understanding of the material, the authors read the transcripts multiple times. In step I of the analysis, meaning units pertaining to experiences of both possibilities and challenges in meeting oral health needs among patients were identified in the transcripts. Subsequently, these meaning units were condensed (step II) and then labelled with codes (step III). The coding process was conducted manually by the first author and iteratively refined and discussed with the co-authors to ensure rigor in the analysis. Following this, subcategories were derived from the codes in an inductive manner with a focus on low abstraction and low interpretation (step IV). Finally, the eight subcategories were organized into three categories (step V) through collaborative discussions until consensus was reached among all authors. This collaborative approach enhanced the trustworthiness of the study. The analysis steps are described in Table 2.

**Table 2:** Examples of the analysis process

Meaningful unit	Condensation	Code	Subcategory	Category
Oh yes (pulls on his yes, at the same time pause for thought/silence) I think it's hard to say a lot about the possibilities oh I can say a lot about the challenges hahaha … Oh, very often it is patients who generally have difficulty orienting themselves in society and we also have a society and welfare that is very divided. You can't get under a roof, but you have to seek care there, there, there and there … Also the psychiatric care, only that part can be in different places. Oh … different, completely different roofs, would have a new entrance… A lot of people have problems making contact, whether it's psychosocial phobia or if it's because you feel so bad that you can't cope or ADHD that you don't remember or recover or (think) or maybe you haven't learned to take care of yourself, it's not at all uncommon … oh (thinks again) oooh (thinks again) it's this thing with, yes it takes a lot, yes now I'm talking about those patients as the patients, the psychiatry patients, so much is expected of them in seeking care themselves, so for some absolutely, some are otherwise well-functioning. But overall… (a little delay) you get to specialist psychiatry. Then it is often more than just a limited depression	Hard to pronounce the possibilities, I can say more about the challenges. It is patients who generally have difficulty orienting themselves in society, our society and welfare that are very divided. Not under the same roof, but care is sought there, there, there and there also the psychiatric part, as long as it can be divided in different places. Different ceilings, different entrances, many people's problems can be to make contact, whether it's psychosocial phobia, or that you feel so bad that you can't cope, or ADHD that you don't remember or recover, or you haven't learned to take care of yourself, it's not uncommon at all… It takes a lot, now I'm talking about the patients, the psychiatry patients, expected to seek care themselves. For some absolutely, some are otherwise well-functioning. But overall… Specialist psychiatry… Then it is more than a limited depression	Knowledge and ability challenge the patient when they live according to the structure and demands of society and organisations	Organizational insight and perceived barriers for patients	Organizational challenges
Because now they have changed the rules. But then nurses could also apply… This is what is called a green card. Certificate of necessary dental care. So if you had… was within the right diagnosis, But now it has become very complicated because now we are not allowed to do it, but it is the doctor and it is like a special place you (laughs a little) the doctor, he becomes, just no.. Because it will be so complicated with this particular application. I think it's a great shame because it takes… In other words, patients will have to wait maybe a very long time. Yes. And I also think that we have a shortage of doctors, there is a shortage of doctors everywhere. And scarce eh… nursing staff at all. And… Eh… The doctors have a lot to do, and then it can… The risk is also that… Applying for a dental certificate, it's like maybe not a priority… Because there are so many other certificates and sick leave, and patients the doctors need to see that take precedence. Because it has been my experience that the doctor has said: Yes, but, it will have to wait, it… do not have the opportunity to do so. And that it's a bit of a complicated system	The rules have changed, in the past, even nurses could fill out the application for a green card, certificate of necessary dental care, with the right diagnosis, More complicated and it is only the doctor who can apply and the doctor answers no because the application is complicated. Which means longer waits for patients. The lack of doctors complicates the situation. Lack of nursing staff at all, doctors have a lot to do, which increases the risk that applications for dental care certificates will have a lower priority. Other certificates, sick leave and other patients take precedence. Heard doctors say, yes but it will have to wait, no possibility and a complicated system	Changed rules for green card, necessary dental care and who can issue certificates	Administrative challenges for the accessibility to dental care	Organizational challenges
(Yes, and the mouth can be one of the intimate body parts) Oh it's hard! But I think that you could also de-dramatize it, you don't have to make it so big with your oral health, you can actually just ask, how is your mouth going? Ah… So when I was studying my specialist education, I wrote my essay about … uhhh… Talk about sexual dysfunction in psychiatry. Ah… uhhh… It was hard to ask about because it's a bit embarrassing and so on, but there were also many who connected it, yes it's very difficult to ask about suicidal thoughts in the beginning as well. Just like that, I think too… We in psychiatry usually say that we are good at looking at the big picture, but we lose that, we don't always do that (Could it be that you have to train yourself on, what kind of battery do you have with answers?) yes! But exactly and what do I do with the answer? I can't say no, then maybe you need to have a way to go… I can help you call the dentist or whatever it may be. Just like that, I think too… We in psychiatry usually say that we are good at looking at the big picture, but we lose that, we don't always do that	Oh it's hard! But I think you could de-dramatize it, you don't have to make it so big with your oral health, you can actually just ask, how is your mouth doing? When I was studying my specialist training, I wrote my essay about talking about sexual dysfunction in psychiatry, it was hard to ask about because it's a bit embarrassing, but there were also many who connected it, yes it's very difficult to ask about suicidal thoughts in the beginning too, it will be hard in the beginning … But you have it in the package as well. There is no difference really. Yes! But exactly and what do I do with the answer? I can't say no, then maybe you need to have a way to go… I can help you call the dentist or whatever it may be. Just like that, I think too… We in psychiatry usually say that we are good at looking at the big picture, but there we lose, we don't always do it	On reflection, it can be de-dramatized, just as talking about sexuality or suicide can be part of the conversation. The question is, what do I do with the answer? We say that we look at the whole picture in psychiatry!?	Multifaceted factors influence oral self-care and dental care	Patients' opportunities to live healthier lives
(Do you think it could be out of ignorance? Or… that it doesn't feel justified? Or what do you think? What is it about?) Yes, yes it's a bit … Actually, it should be just as important as with the body and with everything else, as well as with it's just like it's an isolated island. hmm… where oral health is not really included so I think it is very important to include it as part of that … I don't know how you think that? Mmm… So I think so and that it will come fairly in time that you can then bring up that, how, that is, concretely like that what is what, yes knowledge-wise, what happens and what does it mean and what does it do for me now and in the future for the rest of my life… It's very good to take care of your teeth heehh… Otherwise, it can be damn expensive and painful in the long run	It is a little outside the body and should be just as important, oral health is not really included. The informant highlights that the mouth should be just as important as the rest of the body, to gain knowledge, what is happening, the importance of oral health now and for the rest of my life and that it can be expensive in the long run	Oral health is excluded even though it should be just as important, knowledge is lacking about the importance of oral health both here and now and what it leads to in the long term	The mouth as an “uncertain”and isolated part of the body	Staff roles and attitudes

### Reflexivity

The authors of this study bring diverse scientific backgrounds and lived experiences to the research process, which both enhance and constrain the understanding and interpretation of the results. Collectively, the group includes experience in qualitative research and the team’s expertise spans psychology, public health, and odontology, with three members being dental hygienists and one being both a medical doctor and a public health scientist. This distribution of competencies has informed the study’s interdisciplinary perspective and contributed to a nuanced analysis of the findings.

## Results

**Fig. 1:**
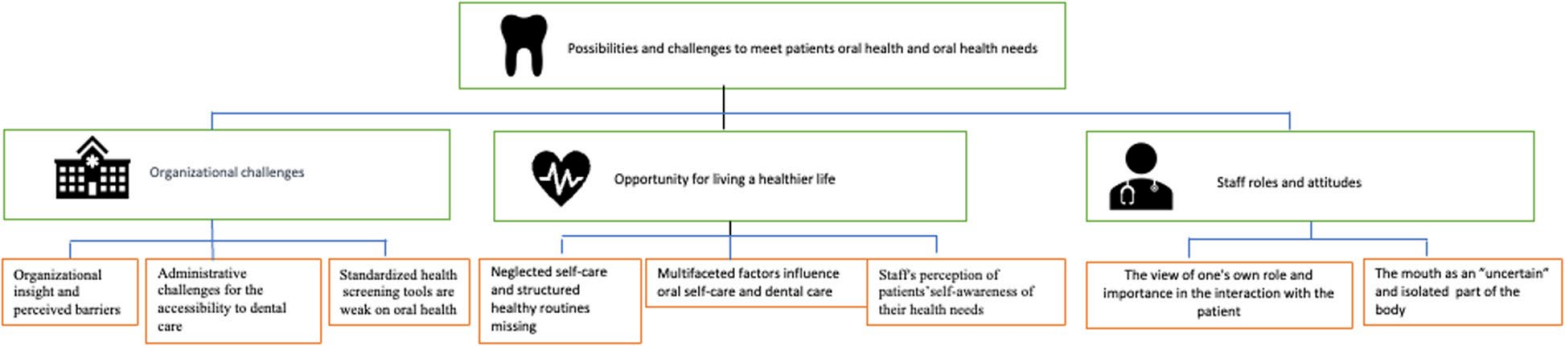
The eight subcategories forming the three categories

Participants in various professions within the field of psychiatry (Table 1), including both licensed and unlicensed personnel, were interviewed with the aim of exploring the experiences of psychiatric professionals regarding the possibilities and challenges in meeting oral health needs among patients, and these were structured from the eight subcategories into three categories: 1) Organizational challenges, 2) Opportunities for living a healthier life, and 3) Staff roles and attitudes (Fig. 1).

### Organizational challenges

This category includes the informants’ views on their workplace and how the psychiatric care is organized for both them and their patients. It is based on three subcategories: *Organizational insights and perceived barriers for patients,* describing the challenges of the organizational structure in psychiatry, especially in regards to regulations; *Administrative challenges for the accessibility to dental care,* including patient access to dental care and administration and rules for patients receiving the mandated dental care subsidies; and *Standardized health screening tools are weak on oral health,* including approaches to getting to know patients and their personalities, strengths, challenges, and needs.

### Organizational insights and perceived barriers

The informants described how psychiatric care in Swedish inpatient and outpatient units is geographically spread out across many locations and buildings in the municipalities. This means that any collaborations that the staff need to have to adequately meet patient needs are complicated. The informants expressed the need to be active in their roles, aware, and competent. The informants reported that there are formally organized collaborations between psychiatric and addiction care services spanning various specialties and including partnerships between physicians and health centres. Some of these collaborations are based on specific agreements. However, some informants expressed an organizational uncertainty regarding which dental clinic they should turn to when a patient requires dental care. Other informants were very familiar with which dental clinic the unit cooperates with in cases where a patient without previous dental contact needs dental care. The informants reported that they feel that the patients are challenged because the healthcare system today expects the patients to have self-awareness, to have an understanding of their own situation and disease, and to be involved in the decisions regarding their treatment and to actively participate in improvement and recovery. One informant expressed frustration:

I think it's hard to say a lot about the possibilities, but I can say a lot about the challenges... Very often it is patients who generally find it difficult orienting themselves in society, and we have a society and welfare system that is very divided. You can't find everything under one roof, and you have to seek care here and there. (Psychologist)

There are many different types of professionals involved in the care and treatment of psychiatric patients (i.e., physicians, psychiatric nurses, nurse practitioners, care aides, psychologists, social workers, etc.), and these are all spread out over different locations and with their own management responsibilities. Connecting with staff in dental care and creating an ongoing contact is an even greater challenge. One informant described the complexity of the organizational divisions as follows:

The challenge is time; currently, we screen and refer to other professionals. We are not supposed to have the complete picture, but for psychotic patients we would need a more comprehensive view. Their difficulty lies in seeking help from various services, such as primary care and dental health. I have worked for a long time, and previously everything was available in one place, and the entire situation was taken care of. (Psychiatric nurse)

An informant expressed thoughts about the lack of a holistic view on the patient within the organization and the need for screening and distributing tasks and responsibilities across different types of units. This can be further complicated by the fact that the mental health of patients can change rapidly.

The informants believed that there is a need for a change in the psychiatric organization from a medical perspective (such as focusing on blood and liver samples) to a focus on the patient as a whole, including their social life. Almost all patients have some form of social problems in their background, which contributes to comorbidity and complexity that cannot be remedied with medication. After a moment's reflection, one informant recalled a conversation in which oral health needs were discussed:

Seldom, barely twice a year, does the dentist come up in conversation, unless one has a toothache, like this, and I have never heard that the request has been granted. Well, I can vaguely recall a conversation where it was mentioned that a patient had trouble with their teeth. So! But I must admit it was a minor issue in the conversation. Patients do not bring toothbrushes, and almost everyone gets one here, and I probably haven't reflected on whether their situation was taken care of other than assuming it was. (Social worker)

### Administrative challenges for the accessibility to dental care

The informants described the administration of subsidies for dental care (afforded by law to patients with illness or disability that entails an increased risk of deteriorated dental health) as both a challenging process and an important opportunity when the patient requires dental care. The informants expressed frustration over the complex organizational administration required in applications for dental care subsidies on behalf of patients, and they emphasized that they struggle to understand the criteria, conditions, and diagnoses that make one eligible for dental care subsidies. This confusion leads to recurring discussions among colleagues, which wastes time and creates frustration and irritation. Unlike before, it is now only physicians who are eligible to submit these applications. This means that doctors are assigned additional administrative tasks, and thus dental care issues, dental care needs, and applications for dental care support tend to be given lower priority:

Now they've changed the rules. Previously, even nurses could apply for certificates for necessary dental care if the patient had the right diagnosis. And now the physician, he just says 'no'. Because it gets so complicated with this application. I think it's such a shame! Because the patients have to wait for a very long time. (Psychiatric nurse)

Despite administrative challenges, informants viewed dental care subsidies as a valuable tool for informing and motivating patients to address dental care needs. They stressed the importance of timely care, and called for reduced costs and improved accessibility. One informant noted that dental care tends to be more accessible in affluent areas, where initiatives like school visits by dental hygienists frequently occur, though such services are not universally available.

### Standardized health screening tools are weak on oral health

The informants often use various screening instruments to learn more about the situations of patients, including their overall health, life and health-related goals, and lifestyle habits, including physical activity, diet, sleep, and psychosocial resources. The staff reported having investigated and mapped patient resources and needs and having planned subsequent health-promoting activities, but oral health is not yet a natural part of psychiatry, which means that the overall picture of the patients’ needs is missing.

Some informants drew indirect conclusions regarding whether oral health is part of their work given that that diet and drinking habits are included in the screening tool. Another informant suggested integrating tooth, mouth, and oral hygiene into patient education, which in turn would require staff training. For example, the informants proposed integrating questions about oral hygiene habits into existing inquiries about diet and exercise. One informant suggested expanding the responsibilities for municipality housing support services to incorporate questions about patient oral hygiene habits, products, and methods. Currently, the focus in housing support services is primarily on the availability of hygiene products at home rather than preventive measures for everyday oral hygiene habits. One informant highlighted the need among staff for more knowledge on how patients can be encouraged to focus on their oral health:

And the dental care questions we have are: Have you seen a dentist or dental hygienist in the past two years? Do you have any issues with your mouth? Do you have bad breath or a bad taste in your mouth? Does your diet, for example, get affected by dry mouth, poorly fitting dentures, tooth loss, or mouth pain? Yes. These are indeed excellent questions, but there's nothing about how or why one brushes their teeth. Much more knowledge is needed. (Psychiatric nurse)

The dialogue between the patient and the staff was described as an important tool where strategies, such as open-ended questions and motivational interviewing methodology, are used to motivate and get to know the patient better. Consistently examining each persons’ unique personality, daily life, behaviours, and potential challenges is important in order to identify appropriate interventions.

### Opportunities for living healthier lives

This category includes the informants’ views on the needs of patients to live in a healthier manner with their mental illness. The category is based on three subcategories: *Neglected self-care and structured healthy routines are missing*, exemplifying the factors for well-being and health-promotion, such as lifestyle choices, behaviours, and daily routines; *Multifaceted factors influence oral self-care and dental care*, including circumstances that contribute to challenges for patients, e.g., socioeconomic status, upbringing, attitudes, and habits related to general and oral health; and *Staff's perception of patients´ self-awareness of their health needs,* highlighting the significance of patient self-awareness of their mental condition and its effects and the importance of building patient motivation for positive change in the midst of their difficulties.

### Neglected self-care and structured healthy routines are missing

The informants reported that lifestyle, habits, and mental health significantly, and often negatively, impact their patients’ overall well-being. Their interpretation is that patients can better manage and improve their mental health with structured daily routines, regardless of whether they are in the hospital or at home. Identifying health-promoting activities was seen as central to developing overall healthy habits. Toothbrushing is believed to be a healthy morning and evening habit. As a reminder, the patient is advised to take their prescribed medications in connection with brushing their teeth as an everyday habit. One informant pointed to the lack of routines to help with, for example, brushing teeth:

The people we meet often have bad everyday routines and disrupted circadian rhythms, like they sleep until four in the afternoon. Morning routines such as eating breakfast and brushing your teeth? No, there isn't. (Municipal housing support aide)

The informants also described variations in eating habits among patients. While hospitals provide regular meals and allow patients to purchase optional snacks and drinks, patients at home often have irregular eating patterns, preferring fast food, snacks, energy drinks, and soft drinks. For some patients, municipal housing support aides play a crucial role in establishing and maintaining a balanced daily life, and they are an important resource when patients lack an understanding of their illness. One informant described it like this:

It is challenging to work with mentally ill patients. They may not think about their physical or oral health at all until they get better or medicated. It's a challenge because you want the patients to feel good physically as well. Taking care of basics, like sleep, diet, exercise, hygiene – the whole thing is difficult. The patient may not think it's that important really, but it's the basis of it all, I think. (Psychiatric nurse)

### Multifaceted factors influence oral self-care and dental care

The informants described how the self-care abilities of patients vary due to both external and internal factors. External factors, such as the patient’s background, socioeconomic condition, culture, and knowledge influence attitudes and behaviours related to general and oral health. The informants reported often hearing patients express that dental care is expensive, and they concluded that the cost of dental care is given lower priority by the patient than, for example, paying rent for their accommodation. One informant perceived that the population-based preventive programs provided by the dental public health service system vary depending on the socio-economic status in different sections of the city or community.

The informants reported that they perceive that patients’ routines for oral hygiene are private and often kept secret. This leads to patients easily experiencing an invasion of privacy when staff ask them about their oral health. The topic can be sensitive and may be compared to topics such as sexuality or suicidal thoughts:

Oh, it's hard! But I think that you could also de-dramatize it; you do not have to make it so big with oral health; you can actually just ask, ‘How is it going with your mouth as well?’ (Psychiatric nurse)

Further internal factors, including shame about their oral health status, hinder patients from seeking dental care. Improved mental health increases patients’ interest in maintaining oral care, e.g., addressing broken teeth in order to reintegrate into society because intact teeth are generally seen as a social marker. One informant pointed to the shame patients experience when their teeth have been destroyed and the joy and happiness patients show when their teeth are repaired.

Informants described patients' fear of oral and dental care as multifaceted. Examples included discomfort with toothbrush bristle material and delusions related to brushing:

I had one who couldn't brush because then, like, like the devil came out of her mouth. So, there are so many delusions around it sometimes. Oh, it's all about finding that stuff. To talk about it. (Psychiatric nurse)

During dental treatments, pain and discomfort can be experienced by the water jets hitting the oral mucosa, or panic from the water or unknown liquid running down the throat. Others find that the fingers in the mouth of the dental staff induce nausea. Some patients are afraid of needles, and some are afraid of simply being probed in the mouth, which complicates dental procedures.

### Staff's perception of patients´ self-awareness of their health needs

In order to succeed with a behavioural change, the informants emphasized the importance of patients´ having insights and knowledge about their symptoms and their condition as well as an understanding how their diagnosis impacts their general health and oral health. Patients tend to lose motivation, perhaps because they lack knowledge or lose the drive to maintain changes during periods of poor health:

The challenges are to find ways for the person to find their driving force for themselves and their health, including everyday life and routines. (Psychiatric nurse)

One informant described the challenge of waiting for the patient to understand and gain the same insight about their illness as the staff, as well as waiting for the patients to recognize what they can do for themselves and their well-being in this way:

I can find it difficult sometimes that these basic things that we can see, they themselves don´t see, they don't understand that their feelings can improve by walking half an hour a day or eating properly; they lack the experience, and their solution is medicine. (Psychiatric nurse)

### Staff roles and attitudes

This category contains the informants' views on their roles, assignments, knowledge, and attitudes as professional caregivers and is based on two subcategories. In the first – *The view of one's own role and importance in the interaction with the patient* – the informants see themselves as professionals who promote and motivate healthy changes in their patients. It also includes their understanding of their mission, both overall and specifically in terms of patients' oral health needs. The second – *The mouth as an isolated part of the body* – includes how the informants view oral health and how they act when they discover that their patients have oral health needs.

### The view of one's own role and importance in the interaction with the patient

The informants described their role and their mission with great commitment. To be able to support and treat patients requires patience and time as well as trust. This represents a relationship that the informants believe takes time to build. In addition, understanding, knowledge, empathy, and respect for the patients´ situations, personalities, and challenges are required, as is seeing the individuals behind the diagnoses. One informant compared their work to "sowing the seeds" of change by supporting and waiting for the patient's insight, motivation, and goal-setting that promote the patient's journey towards a healthier life:

It's about strengthening my relationship with the patient so they can trust me. Yes, I believe it's through time and nurturing the relationship, showing interest, and allowing it time to develop. It's about time and showing that I'm willing to listen. (Psychiatric nurse)

Informants highlighted their responsibility to consider patients' medical histories and broader aspects of their well-being. They noted that oral health could be more effectively addressed through alternative methods. One informant emphasised the importance of genuinely exploring patients' attitudes, knowledge, and habits, such as oral hygiene, to better understand their individual needs.

### The mouth as an “uncertain” and isolated part of the body

The informants described how they actively help and support patients to seek dental care when the need arises. They expressed a feeling of uncertainty when it comes to detecting the needs of the patients unless the patients themselves explicitly state their needs, and the informants stated that they were unsure of how to advise their patients on how to take care of their oral health. The informants believed that their own knowledge, insights, and attitudes greatly influenced and determined how they integrated oral health into patient care. On the other hand, they said that if the patient experienced oral dryness (xerostomia) from medications, they are often suggested to counteract their oral dryness with saliva-stimulating products. Some informants stated that they were aware, confident, and knowledgeable about how bacterial coatings are identified on the patients' teeth, while other informants stated that they were uncertain and expressed a need for more knowledge. Some informants were curious and asked questions about the effect of snuff on gums, dental health, and general health. One informant described how the oral cavity is like an isolated island from the rest of the body:

It should actually be as important as with the body and everything else, it's just like it's an isolated island, oral health isn't really included, so I think it's really important to bring it in. Yes, in terms of knowledge, one should know what's happening and what oral health means, and what it does for me now and in the future for the rest of my life, it's very good to take care of your teeth, otherwise it can get very expensive and painful in the long run. (Psychiatric nurse)

## Discussion

This study aligns with prior research indicating an organizational gap between psychiatry and dentistry [1, 3–5, 37], and the results of this study revealed a variety of challenges and opportunities. The challenges involve organizational issues, complex administration, and the absence of strategic tools for oral health integration. The informants also experienced that patients face daily hurdles affecting their overall well-being. Opportunities arise from integrating oral health into existing tools and fostering collaboration between staff in psychiatry and dentistry in order to prevent and promote healthy lifestyle changes.

In the organizational structure of Swedish psychiatry, the informants described how providing dental care alongside psychiatric services presents both challenges and opportunities, especially when integrating patients’ dental needs with their psychiatric treatment. Dental care has also been described as lacking in the existing integrated patient-centred process in healthcare, and there is a lack of a structured framework for collaboration [1, 3, 5]. Swedish healthcare legislations emphasize meeting the healthcare needs of all residents equally, and that those with greater needs should be prioritized [27, 38–40]. The Swedish laws that regulate forensic psychiatry [40] and patient safety [41] require patient participation, while the Swedish Social Services Act [42] emphasizes people's right to self-determination and integrity, and these laws need to be used in combination to fit patients’ needs.

Our informants reported that patients’ oral health was negatively affected by long-term medication, lack of family support, limited dental care access, insufficient oral hygiene guidance, and financial constraints. They also experienced patients' unwillingness to cooperate, refusal of care, communication difficulties, resistance to mouth opening, reluctance to brush, and limited activity resources, and this confirmed the results of a Swedish systematic evidence map on the associations between oral health and mental health [5]. It is suggested that these experiences be integrated into existing screening tools and patient-centred healthcare processes.

Screening and examining patients’ symptoms, medical conditions, and social situations is a truly central task, and in the collected subjective and objective data presented here it appears that patients are often referred to relevant professionals in psychiatry care and/or in health care. Our findings indicate that staff desire a holistic approach to patient care, which is hindered when practices are fragmented. Also, the results show that SMI profoundly affects patients’ overall health and social situation. Our results echo previous research, emphasizing the need for education and appropriate tools to effectively integrate oral health into patient care [3, 15, 23, 29, 43], especially into psychiatric care [5].

The informants experienced those patients with SMI neglect daily healthy routines, including dental hygiene, eating habits, physical activity, and sleep, which affects both oral health and mental health. Previous studies confirm this neglect [24, 44], showing the importance of considering daily routines and habits from both an oral and mental health perspective. Our results show a curiosity and willingness to look at the patient’s whole picture and where the informants have thought about the importance of oral health for general health and well-being. Toothbrushing is considered a natural part of the patients' daily routines, but without actually checking whether the patients actually have the habit of brushing their teeth twice a day. On the other hand, staff often view patients’ oral hygiene routines as private, and patients tend to keep them secret. Combining this study with previous research, it appears that collaboration and knowledge exchange facilitate the continued development of evidence-based programs, benefiting both the patient's overall and oral health [36].

Patients may keep oral hygiene habits secret from psychiatric staff due to a perceived division between psychiatric and dental care, believing that no dental help is available. Open questions about oral health could bridge this gap. However, one informant compared discussing oral health to sensitive topics, like sexuality or suicidal thoughts, emphasizing the issues of personal integrity related to such inquiries. Additionally, shame about dental issues has also been described as a factor in not talking about oral health concerns [10].

The informants noted that improved mental health increases patients' interest in oral care, and these benefits have been shown in previous studies [1, 10]. Our informants highlighted that broken teeth can motivate patients to seek care in order to reintegrate into society because intact teeth are seen as a marker of social status [5]. Research indicates that individuals with SMI often have coexisting medical conditions and stigma, including oral health issues, which impacts their well-being, self-esteem, social interactions, and daily activities [10, 19, 22]. We argue that integrating oral health into existing person-centred interventions would enhance the holistic understanding of the patient's situation, as demonstrated by previous studies [17, 31, 45].

Dental care subsidies work as initial support for patients and can act as a motivator, especially for patients needing more than emergency dental care. However, staff encounter challenges due to complex administrative procedures for determining eligibility. This problem is also acknowledged in the SNBHW’s evaluation of the dental care subsidy from 2018 [45]. The informants reported positive organisational benefits when the psychiatric ward has a clear collaboration with, and knowledge of, which dental clinic can accommodate their patients. There is a global recognition of vulnerable groups, such as SMI patients, where the WHO proposes that mental health services should be integrated into public health care in order to ensure sufficient financing and that funds must be allocated during the planning for community-based, culturally sensitive, and cost-effective activities [12]. Prior research on oral health underscores the need for improved coordination and continuity of care, especially for complex cases where oral health is often overlooked [31].

Current national guidelines place the responsibility for cooperation between psychiatry and dental care on dental professionals [46], highlighting a gap in addressing oral health within guidelines for depression, anxiety, and schizophrenia-like conditions [36, 46]. Psychiatric professionals often lack an awareness of oral health’s impact on overall well-being [47], and in support of this our informants were unaware of how oral diseases can exacerbate general health conditions, especially in individuals with systemic diseases like diabetes or cardiovascular disease. Dental professionals have also expressed the need for more knowledge about patients with SMI [1, 4], indicating the importance of collaboration between dental and psychiatric care and staying updated with guidelines, even those outside one’s own area of expertise.

Further, our data indicate that municipality housing support services, care aides, psychologists, social workers, and occupational therapists, regardless of their role, can play important roles in patients’ oral hygiene habits, and therefore expanding the responsibilities to include inquiries about oral hygiene habits, products, and methods could enhance patients’ overall well-being. Previous research indicates that psychiatric nurses play a key role as health collaborators [26, 48]. In our study, we observed not only psychiatric nurses, but also other staff members who could incorporate oral health perspectives into their work. Our findings suggest that integrating oral health components into standardized health screening tools and patient education materials could be a beneficial initiative. Additionally, a national systematic review highlights the benefits of enhanced collaboration and competence through the involvement of additional stakeholders such as the social services [5]. The content of this study is suggested to be used in developing psychoeducational models within psychiatry.

One informant described the oral cavity as an isolated island within the body, a perspective that aligns with Andrew Abbott’s framework in *The System of Professions* (1988). Abbott explains how professions establish, defend, and sometimes lose control over their "jurisdictions"—areas of specialised expertise. This notion of jurisdictions being technical (skills), social (public legitimacy), or legal (formal rights) resonates with how dentistry historically emerged as a distinct profession, separating the mouth from the rest of the body. Professions adapt, compete, or relinquish control in response to external forces such as technological advancements, regulations, and societal changes. Abbott underscores that professions maintain their authority by asserting exclusive knowledge through specialised education and institutional legitimac[49].

For example, informants in the current study noted that medications can negatively impact patients' saliva production. They recommend saliva stimulants and sometimes replace medications to boost saliva production, indicating a lack of preventive knowledge. Initially, advice should be given on stimulating natural saliva production before adding drugs and saliva-stimulating products.

Supporting patients in caring for themself and adopting behaviours to prevent more illness can promote and maintain good health [50]. Thus, the results of this study can support the need for developing a psychopedagogical model that integrates oral, mental, and overall health and reduces some of the gaps between psychiatry and dentistry. In the Swedish national guidelines for schizophrenia, psychoeducational interventions like the Illness Management and Recovery program are proposed, focusing on self-management, goal setting, and recovery skills [51, 52]. However, oral health is not yet included in such programs.

Overall, this study emphasizes the need for collaboration, education, and strategies that include oral health perspectives in both existing and new pshycopedagogical models without overloading an overburdened staff in an already complex patient situation.

### Strengths and limitations

Strengths and trustworthiness were ensured by including informants with diverse work experiences, professions, education levels, genders, and ages, thus providing a broad range of insights into patients' oral health needs. Strengths of the study also include the flexibility of conducting interviews via digital platforms or mobile phones, thus reducing wait times and workday interruptions. The digital approach enabled nationwide interviews, minimizing environmentally impactful travel. Recruitment through the snowball effect indicated that informants had relevant experiences, enhancing the study's credibility. The use of an interview guide contributed to dependability. However, phone interviews limited the ability to observe body language, and using a central administrator in Sweden might have impeded information dissemination. Collegial consultation during the analysis process increased the study's confirmability [53].

## Conclusions and practical implications

Psychiatric staff possess central knowledge and insight into the life situations of patients with SMI, and psychiatric staff consider dental staff to be key partners. Collaboration between psychiatric and dental staff is essential in developing strategies to integrate oral health perspectives into current screening and psychopedagogical models and practices. Future research should include patients' perspectives on the possibilities and challenges to meeting their oral health needs.

## Supplementary Information

Supplementary Material 1.

## Data Availability

No datasets were generated or analysed during the current study.

## Abbreviations

SNBHW: National Board of Health and Welfare
SMI: Severe mental illness
SALAR: Swedish Association of Local Authorities and Regions
WHO: World Health Organisation
