# Supplementary material for: Oral health and oral health needs among patients with serious mental illness: reflections and experiences of psychiatric staff in Sweden
Source: BMC Psychol. 2025 Apr 28;13:446. doi: 10.1186/s40359-025-02780-3 (PMC12036200; doi:10.1186/s40359-025-02780-3)
Supplement: Supplementary file 1 — Supplementary Material 1. [file 40359_2025_2780_MOESM1_ESM.docx]

### Interview guide to staff in psychiatry settings

## Survey - staff

**Introduction**

**Background questions:**

1. When are you born?

1940-1945 □

1946–1950 □

1951-1955 □

1956-1960 □

1961-1965 □

1966-1970 □

1971-1975 □

1976-1980 □

1981-1985 □

1986-1990 □

1991-1995 □

1996-2000 □

1. Are you?

Woman □

Man □

Other □

1. Enter your highest education

Elementary school □

High school □

College/University □

1. What profession(s) do you have? (More options may be relevant)

Psychiatrist □

Nurse □

Assistant nurse □

Nurse practitioner/practical nurse □
Nurse aides/personal care assistant □

Personal care worker/Housing supporter □
Psychologist □

Counselor □

Occupational Therapist □

Other □

1. For how long have you worked in psychiatry?

1-5 years □

6-10 years □

11-15 years □

16-20 years □
21-25 years □

26 - 30 years □

31-35 years □

36-40 years □

41-45 years □

46-50 years □

## Semi-structured interview guide - staff

*First of all, I want to start by thanking you for letting me meet with you.*

*The purpose of this study is to understand your professional experience as it relates to your the dental and oral health of your patients.*

*Do you have any questions before we start?*

*I would like to start by asking you a general question about your work*

**General information about the work**

Can you tell us about a typical day at work?

Can you elaborate on this...?
 What/How do you mean...?
 You mentioned that....can you tell me more....?

Challenges, support, encourage, manageability

*Now, I will ask questions about the patients' teeth, mouth and dental care, habits, lifestyle and potential connections to the patients' general health*

How do you view the relationship between patients' general health and oral health?

Can you elaborate on this...?
 What/How do you mean...?
 You mentioned that....can you tell me more....?

Can you tell us about an event/situation where a patient needed to seek dental care?

Can you elaborate on this...?
 What/How do you mean...?
 You mentioned that....can you tell me more....?

Can you tell us about any event/situation when you have reflected on the patient's mouth and dental status?

Can you elaborate on this...?
 What/How do you mean...?
 You mentioned that....can you tell me more....?

Challenges for the patient, support, encourage, manageability

**Patients' self-care**

What is your overall experience of how your patients take care of their mouth and teeth?

Can you elaborate on this...?
 What/How do you mean...?
 You mentioned that....can you tell me more....?

**Experiences of patients' oral health**

What is your experiences of patients' abilities versus challenges for good oral health?

Can you elaborate on this...?
 What/How do you mean...?
 You mentioned that.... can you tell me more....?

Challenges self-care, support, encourage, manageability

*Diet, drinks, drugs, and tobacco use affect oral health in different ways, there are now some questions that focus on that.*

**Patients' diet and drinking habits**

What is your experiences regarding your patients' diet and drinking habits?

Can you elaborate on this...?
 What/How do you mean...?
 You mentioned that....can you tell me more....?

Challenges diet, support, encourage, manageability

**Experiences of tobacco use**

What is your experience of the patients' consumption and use of tobacco and drugs?

Can you elaborate on this...?
 What/How do you mean...?
 You mentioned that.... can you tell me more....?

Can you give examples of what motivated the patient to quit smoking and/or stop using drugs/stimulants?

Can you elaborate on this...?
 What/How do you mean...?
 You mentioned that.... can you tell me more....?

Challenges, support, encourage, manageability

**Other**

Is there anything you would like to add in addition to what we have talked about?

 Supplementary questions;
 Based on the above questions, follow-up questions such as;
 Can you elaborate on this...?
 What/How do you mean...?
 You mentioned that.... can you tell me more....?

*Finally; How did you feel answering the questions I asked?*

*Contact information if you wish to contact me and complement your answers.*

*Charlotte Johansson
Karlstad University
Mobile number;
E-mail address;*
